# Closing the eye-tracking gap in reading research

**DOI:** 10.3389/fpsyg.2024.1425219

**Published:** 2024-06-03

**Authors:** Bernhard Angele, Jon Andoni Duñabeitia

**Affiliations:** Centro de Investigación Nebrija en Cognición (CINC), Facultad de Lenguas y Educación, Universidad Nebrija, Madrid, Spain

**Keywords:** eye movements and reading, reading, eye movements, eye tracking, equality and diversity

When non-invasive, infrared-based eye-tracking technology combined with real-time data processing first became available in the 1970s (Young and Sheena, 1975; Evans and Gutmann, 1978) it opened up a new way of observing cognitive processing in action. Modern eye-tracking has been used in many areas of cognitive psychology, cognitive science, and neuroscience, but it has been especially important in the field of reading research. It seems fair to say that more has been learned about information processing and eye movement control in the past 40 years since the introduction of infrared-based eye-tracking than in the 100 years of reading research before that, starting with the first experiments in 1879 (Huey, 1908; see also Wade and Tatler, 2008). These advances have led to the development of computational models of word identification and eye movement control during reading, such as E-Z Reader (Reichle et al., 1998, 2003, 2009), SWIFT (Engbert et al., 2002, 2005; Kliegl and Engbert, 2003; Schad and Engbert, 2012), Mr. Chips (Legge et al., 1997), the Rational Model of Reading (Bicknell and Levy, 2010; Duan and Bicknell, 2020), and, most recently, OB1-Reader (Snell et al., 2018) and Chinese Reading Model (Li and Pollatsek, 2020). However, a closer examination of the publications on eye-movement research during reading reveals surprising disparities in terms of the countries where eye-tracking research is taking place.

## Publications on eye-movement research in reading

**Figure 1:**
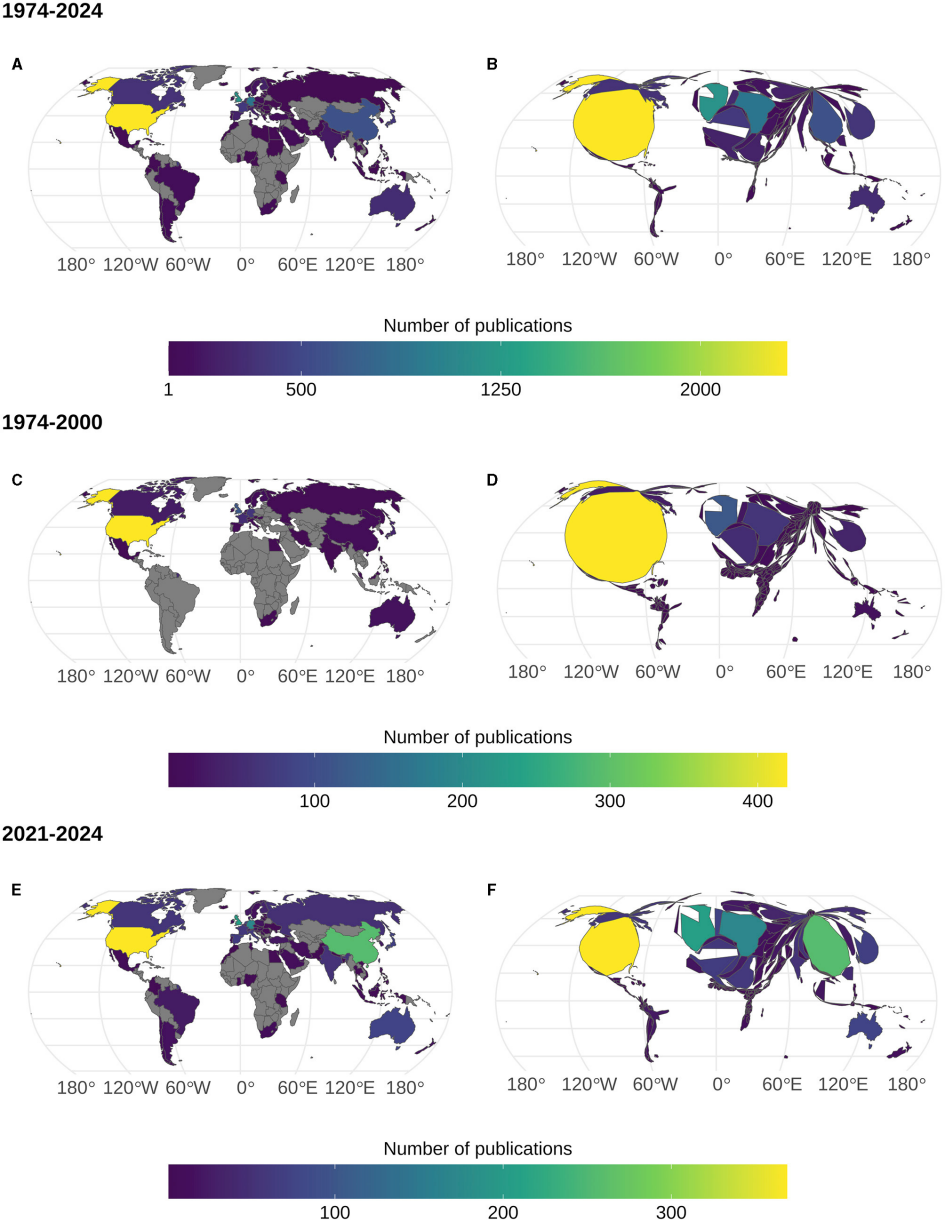
Author's country affiliations of publications on eye-movements in reading by time period. **(A, B)** show the entire period from 1974 to April of 2024. **(C, D)** show the initial phase of eye-movement research in reading from 1974 to 2000, where the United States dominate. **(E, F)** show the most recent period from 2021 to April 2024, where eye-movement research is more international, but still mainly takes place in a handful of countries. (A, C, E) are choropleth maps with number of affiliations represented by color. **(B, D, F)** are cartograms showing the same data as the choropleths in which country boundaries are additionally distorted such that their area is proportional to the number of author affiliations. Figures created with *ggplot2* (Wickham, 2016) and *cartogram* (Jeworutzki, 2023). Figure R code and data available at https://osf.io/rcd9g/.

In April 2024, Scopus (Elsevier) listed 7,473 documents (mainly journal articles and conference proceedings) published in the last 50 years related to eye-movements in reading^1^. When split by country affiliations of the authors, this corresponds to 9,320 country affiliations (one publication could have authors affiliated with more than one country and 271 unclear affiliations were excluded). Out of these affiliations, 2,326 (or 24.3%) corresponded the United States, followed by the United Kingdom (1,195 or 12.5%), Germany (902 or 9.4 %), and China (604 or 6.3%). If we aggregate the results by continents, 47.3% of author affiliations (4,331) were based in Europe, 29.2% of affiliations (2,670) were based in North America, and 18.2% of affiliations (1,663) were based in Asia (apart from China, these publications came mostly from Japan and South Korea). Oceania had 3.39% (310) of publications (of which most were based in Australia and New Zealand). Very few publications had affiliations with authors outside of these areas, with only 64 affiliations (0.7%) from Africa and 116 (1.27%) from South America. Figures 1A, B shows this inequality. Comparing the situation in the first 25 years (1974–2000, Figures 1C, D) with the situation in the last 3 years (2021–2024, Figures 1E, F) this pattern has hardly changed, with one major exception: In the first 25 years, China did not play a major role in reading research using eye movements, but, since 2021, China's contributions (257 affiliations) have only been surpassed by the United States (369). This clearly reflects the massive investment in reading and eye-movement research in China over the last 20 years. Unfortunately, apart from China's enhanced role in eye-movement research, little else has changed: In the past 3 years, most eye movement research still took place in the same Western countries that had dominated the field in 1974–2000.

## Eye-tracking should be truly global

There are several reasons to pursue a more global approach in the use of eye-tracking research reading. Relying only on results from participants from a scarce number of specific countries harms the field. As Henrich et al. (2010b) pointed out, participants from Western, Educated, Industrialized, Rich, and Democratic (“WEIRD”) countries are not representative of all of humanity (see also Henrich et al., 2010a,c; Schulz et al., 2018). Therefore, drawing conclusions about language processing and reading in general, based only or mostly on results from such participants, is difficult to justify (Andringa and Godfroid, 2020).

Since reading inherently implies a (portion of a) text in a particular language, with its own properties and writing system, notions of “generalized” or “universal reading” are necessarily limited (cf. Rueckl et al., 2015). Reading Up to the early 2000s, reading research was essentially research on reading in English. When Keith Rayner published his highly influential review article (Rayner, 1998), most of the results were on reading in English with few results from other languages. How many important results have we been missing due to limiting ourselves to such a short list of languages? We will not know what proportion of findings we thought to be universal just happen to be common to the languages on this list until we start studying reading in all languages people use to read and write. This could enable global replication efforts similar to #EEGManyLabs (Pavlov et al., 2021), with researchers all over the world collaborating on replicating key effects.

## Reasons for the eye-tracking gap

Why is eye-tracking not used more around the world? In theory, apart from behavioral methods, eye-tracking is the ideal technique to use in order to study language in laboratories with limited resources. Unlike EEG, MEG and fMRI, which have extremely high set-up and/or maintenance costs, once an infrared-based eye-tracking system is set up, it requires only electricity to function. Additionally, modern compact eye trackers are completely self-contained and can be set up easily in any environment where a typical laptop computer can be used.

Despite this, as we can see from the publication statistics, eye tracking seems to be used very little to study reading outside of Western and East Asian countries. Why is this? The answer lies almost certainly in the high initial cost of purchasing an eye-tracking system, which, depending on the model and manufacturer, is somewhere between 3,000 and 50,000 US$. Not all eye-trackers are equally suited for reading research, however. Most reading studies are performed with eye-trackers that have a sampling rate of at least 500 Hz. In the last 15 years, recording eye-movements at 1,000 Hz or even 2,000 Hz has become the de-facto standard (e.g., Kliegl and Laubrock, 2017). For many researchers in countries with limited resources, buying such expensive eye-trackers is simply impossible.

In past years, there have been some promising attempts at creating more affordable eye-tracking solutions such as the Eye Tribe eye-tracker (Ooms et al., 2015). However, the Eye Tribe was acquired by Oculus VR/Meta in 2016, stopping development of the product. Similar acquisitions also happened with other companies (e.g., SMI, which was acquired by Apple). Unfortunately, this has, so far, not resulted in devices with research-level eye-tracking capabilities (cf. Valliappan et al., 2020, but this technology has not been made publicly available). Consequently, there are few alternatives for a researcher who does not have the funds for buying a high-precision eye-tracker.

Online eye-tracking using webcams is emerging, with Webgazer.js (Papoutsaki et al., 2016) being the most prominent example. However, using the participant's device naturally comes at the cost of precision and introduces new complications such as accounting for the effect of head movements, screen size, environmental illumination, etc. While there are some encouraging results from recent studies on non-reading tasks (e.g., Slim and Hartsuiker, 2023; Steffan et al., 2024), online eye-tracking has not been formally tested for reading research, since the available sampling rates are extremely low (most webcams can record a maximum of 30 or 60 images per second). In a previous study (Angele et al., 2022), we showed that even behavioral experiments with precise timing requirements can be replicated online. If the same is true for eye-tracking, it would be ideal for enabling eye tracking online all over the world. It is, however, clear that, given the limitations in accuracy (Papoutsaki et al., 2016), the experimental designs possible with this technology will be quite limited.

## Discussion

### How can we close the eye-tracking gap?

In summary, there are few options for researchers with limited resources who would like to study language processing during reading using eye-tracking. We propose a number of measures, both at the institutional level and at the level of individual research projects:

1. *Fund initial investment to make existing eye-tracking systems available in more countries*

First, research funders, especially those with a focus on international collaboration and research development, should prioritize projects that make eye-tracking technology accessible in more countries. The simplest way of doing this would be to fund projects that enable researchers in countries with limited resources to make the initial investment to buy a high-precision eye-tracker. The fact that eye-trackers only need a small laboratory space and electricity to work means that this initial investment could provide payoffs for decades to come. This would be the fastest way to enable researchers to use high-precision eye-tracking. Individual researchers in WEIRD countries could contribute to this by establishing collaborations with partners in non-WEIRD countries, applying for grants in WEIRD countries to secure funding for purchasing high-precision eye trackers for non-WEIRD research partners, and committing to work with their new partners to publish research from this new WEIRD-non-WEIRD collaboration.

2. *Fund development of open-source eye-tracking systems*

In general, leaving eye-tracker development to private companies has been very successful and has led to the availability of mature, highly reliable and precise eye-tracking systems. However, it has not led to the development of lower-cost eye-tracking technology that would be accessible to researchers in countries with limited resources for research funding. Additionally, as the acquisitions of the Eye Tribe and SMI have shown, there is always the danger of privately owned technology to be removed from the market and to become inaccessible to researchers. We believe that there is room for the co-existence of both the premium, high-precision devices that are available on the market today, which are easy to set up and whose manufacturers provide excellent support to those researchers who can afford them, and for a less expensive open-source solution that may not offer the same degree of precision (but sufficient to do reading research) and would rely on researchers to solve problems themselves.

3. *Validate existing systems for reading research*

What can individual researchers and research groups do to close the eye-movement gap? For most, developing a new eye-tracker is outside of our area of expertise. However, researchers fortunate enough to have access to a high-precision eye-tracking system can use it to validate lower-precision eye-tracking systems, including online eye-tracking solutions. There will inevitably be limits in terms of what phenomena in reading can be observed using eye-trackers with lower precision, but it may well be possible to observe effects of processing on eye-movements that are numerically large and relatively stable. Validation studies already exist (Ehinger et al., 2019; Kaduk et al., 2023), but not for reading research. Publishing such validation studies will encourage researchers with limited resources to invest in eye-trackers that have been shown to be useful in reading research, and, at the same time, will make it easier for such researchers to publish results obtained using already-verified devices.

## Conclusions

Publication metrics clearly show a gap between countries where eye-tracking research on reading is done extensively and those where such research is virtually inexistent. The example of China demonstrates that, with sufficient investment on the part of research funders, it is possible for a country to move from the latter group to the former relatively quickly. However, this is likely not an option for many other countries given the cost of initial investment necessary to acquire eye-tracking equipment. As a result, the lack of eye-tracking research in most countries and on most languages threatens the validity and generalizability of reading research overall and reduces its overall usefulness. Funding collaborations involving the deployment of existing high-cost eye-tracking systems in countries with limited resources, funding the development of lower-cost, sustainable open-source solutions for eye-tracking, and, on the level of individual researchers, validating existing low-cost eye-tracking devices against high-quality and high-precision eye-tracking systems of reference may, in the future, contribute to making not just reading research, but also other research that relies on eye-tracking more globally available and more useful and insightful for everyone.

## Author contributions

BA: Conceptualization, Writing – review & editing, Writing – original draft, Visualization. JD: Writing – review & editing, Conceptualization.

## Footnotes

^1^Scopus query: TITLE-ABS-KEY (reading AND ((eye AND track^*^) OR (eye AND movement) OR (eye AND movements))).
